# Chitosan Coating Enhances the Antimicrobial Activity of *Punica granatum* L. Phenolic Compounds

**DOI:** 10.3390/life15121878

**Published:** 2025-12-08

**Authors:** Kazim Sahin, Sena Sahin Aktura, Ilkay Bahceci, Zihni Acar Yazici, Burak Oskay, Nebahat Ejder, Emine Yurteri, Derya Bal Altuntas

**Affiliations:** 1 Department of Medical Microbiology, Faculty of Medicine, Recep Tayyip Erdogan University, Rize 53020, Turkey; kazim.sahin@erdogan.edu.tr (K.S.); ilkay.bahceci@erdogan.edu.tr (I.B.); zihni.yazici@erdogan.edu.tr (Z.A.Y.);; 2 Department of Animal Health and Breeding, Provincial Directorate of Agriculture and Forestry, Rize 53020, Turkey; 3 Department of Field Crops, Faculty of Agriculture, Recep Tayyip Erdogan University, Rize 53340, Turkey; 4 Department of Bioengineering, Faculty of Engineering and Architecture, Recep Tayyip Erdogan University, Rize 53100, Turkey

**Keywords:** *Punica granatum*, chitosan nanoparticles, MIC, antimicrobial activity, MTT, cytotoxicity

## Abstract

The development of antibiotic resistance has become a global health challenge, resulting in approximately 800,000 deaths per year. The rapid rise in multidrug-resistant (MDR) pathogens has prompted an urgent need for antimicrobial alternatives. *Punica granatum* L. peel has long been valued for its rich bioactive polyphenols with potent antimicrobial properties. In this study, *P. granatum* L. peel extract (PGPE) was integrated with chitosan nanoparticles (CH-PGPE) to enhance antimicrobial efficacy while minimizing potential cytotoxicity. The antimicrobial potential of PGPE and CH-PGPE was evaluated with agar well diffusion, disk diffusion, and minimum inhibitory concentration (MIC) analyses against standard ATCC and clinical MDR strains of *Escherichia coli*, *Klebsiella pneumoniae*, *Pseudomonas aeruginosa*, and *Staphylococcus aureus*. MTT assay evaluated the biocompatibility and anti-proliferative potential of PGPE on ARPE-19 (normal retinal pigment epithelial), HeLa (human cervical cancer), and A549 (human lung carcinoma) cell lines. PGPE exhibited antibacterial activity, and CH-PGPE reduced MIC values by approximately two-fold. Both PGPE and CH-PGPE demonstrated comparable or superior inhibition compared to several conventional antibiotics, particularly against MDR strains. The MTT assay revealed that PGPE was non-cytotoxic to normal ARPE-19 cells, while exhibiting the highest antiproliferative potency against A549 cells and a moderate inhibitory response in HeLa cells. The nanoparticle-supported formulation enhanced the antimicrobial efficacy of PGPE and also exhibited selective anti-proliferative activity against cancer cells without affecting normal cells.

## 1. Introduction

Antibiotics are the most commonly used drug group in the world [1]. The World Health Organization (WHO) defines the concept of ‘appropriate drug use’ as ‘the ability of individuals to obtain the appropriate drug for their clinical findings and characteristics, at the appropriate duration and dosage’ [2]. However, widespread and improper antibiotic use has led to bacterial resistance, creating a global threat [3]. Diseases caused by resistant bacteria pose a serious health risk, particularly for immunocompromised patients in intensive care, leading to prolonged hospitalization, complications, and increased mortality and morbidity [4]. The decreasing efficacy and growing contraindications of synthetic drugs have increased interest in natural therapies [5].

The medicinal use of plants dates back to early human history [6]. After Stephen DeFelice introduced the term ‘nutraceutical’ in 1989, combining ‘nutrition’ and ‘pharmaceutical’, research on herbal and functional foods gained importance [7]. *Punica granatum* L. (pomegranate), belonging to the *Punicaceae* family, is a nutritionally rich fruit with diverse bioactive compounds, especially polyphenolics [8,9,10]. *P. granatum* has antioxidant, antimicrobial, anti-cancer, and anti-inflammatory properties [11,12,13]. The antibacterial activity of pomegranate exhibits a broad spectrum against both Gram-positive and Gram-negative bacteria, including *Streptococcus mutans*, *Escherichia coli*, *Pseudomonas aeruginosa*, *Klebsiella pneumoniae*, *Listeria monocytogenes*, *Salmonella* species, *Mycobacterium tuberculosis*, β-lactamase-producing *Klebsiella pneumoniae*, methicillin-sensitive *Staphylococcus aureus* (MSSA), and methicillin-resistant *S. aureus* (MRSA) [14,15,16]. The antimicrobial potential of *P. granatum* is primarily attributed to the high content of hydrolysable polyphenols, which can disrupt bacterial plasma membranes and inhibit antimicrobial resistance-related proteins [14,16].

**Figure 1:**
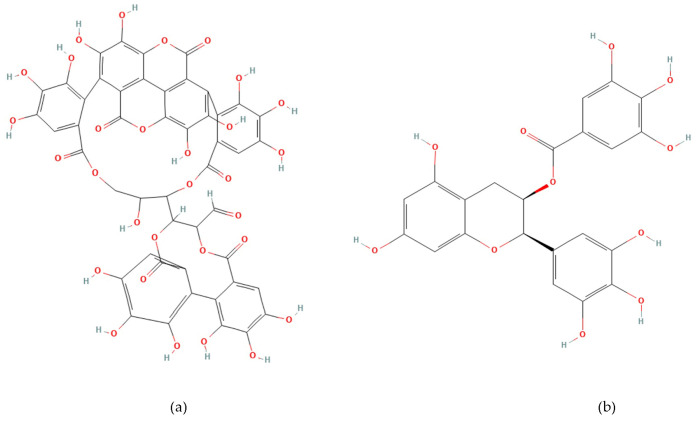
Chemical structure of Punicalagin (**a**) and Epigallocatechin gallate (**b**).

Phenolic compounds are plant-derived secondary metabolites beneficial to human health [17,18]. Punicalagin (2,3-(S) hexahydroxydiphenoyl-4,6-(S,S)-gallagyl-d-glucose, PUN) (Figure 1a) is the most hydroxylated ellagitannin in the pomegranate peel [19,20]. PUN disrupts bacterial redox balance and inhibits “quorum sensing”, the communication system regulating virulence. Interfering with these pathways weakens bacterial defenses, reduces biofilm formation, and enhances susceptibility to antibiotics [21,22]. Epigallocatechin gallate (Epigallocatechin 3-O-gallate, EGCG) (Figure 1b) is a catechin molecule having a gallate group at position 3 [23]. EGCG binds to the lipid bilayer of bacterial membranes, increasing permeability and causing cell death [24]. EGCG also inhibits vital enzymes like DNA gyrase and suppresses protein synthesis [22,25]. The numerous hydroxyl (-OH) groups in PUN and EGCG confer strong antioxidant and free radical scavenging activity, while exhibiting antimicrobial effects by disrupting bacterial cell wall integrity or inhibiting enzymatic processes [23,24,25,26,27].

Nevertheless, the bioavailability of phenolic compounds is often limited due to their chemical structure. To address the limitation, nanoparticles (NPs) are widely used in biotechnology, pharmacology, and medicine due to their unique physicochemical properties, which enable them to adhere to cell surfaces and be taken up into cells through various mechanisms. The conjugation of NPs obtained from natural materials with plant extracts has gained momentum lately [28]. Chitosan is a polysaccharide obtained by deacetylation of chitin, which carries a positive charge, providing strong affinity for negatively charged phenolic compounds [29].

This study aims to isolate phenolics from *P. granatum* and coat the raw extract with chitosan to enhance the stability and antibacterial activity of the compounds, creating a cost-effective and low-toxicity antimicrobial agent.

## 2. Materials and Methods

### 2.1. Materials

HPLC sample analyses were performed using a LC-2030C-3D (Shimadzu, Kyoto, Japan) device, with a diode array detector (DAD) (Shimadzu, Kyoto, Japan) and an RP-18 reverse phase column (Shimadzu, Kyoto, Japan). Methanol (Merck KGaA, Darmstadt, Germany) was used as the extraction solvent, while acetic acid was purchased from Merck KGaA (Darmstadt, Germany) for encapsulation procedures. Chitosan, Tween 80, and sodium tripolyphosphate (TPP) were purchased from Sigma-Aldrich (St. Louis, MO, USA). RPMI-1640 medium was obtained from Capricorn (Düsseldorf, Germany), and fetal bovine serum (10% FBS), along with penicillin-streptomycin (1%, 10,000 U/mL), were purchased from Gibco (Carlsbad, CA, USA) for cell culture.

Reference strains: *Escherichia coli* (ATCC 25922), *Klebsiella pneumoniae* (ATCC 13883), *Pseudomonas aeruginosa* (ATCC 27853), and *Staphylococcus aureus* (ATCC 43300) were obtained from the American Type Culture Collection (ATCC). Clinical isolates were collected from patients at Recep Tayyip Erdogan University Training and Research Hospital (Rize, Turkey). A549 (human lung carcinoma), HeLa (cervical carcinoma), and ARPE-19 (retinal pigment epithelial) cell lines were obtained from the Department of Medical Microbiology, Recep Tayyip Erdogan University.

### 2.2. Preparation of Punica granatum L. Peel Extract

The *P. granatum* L. used in the study was obtained from a local farmer who does not use pesticides (Adıyaman, Turkey). The fruit peels were dried in an oven at 40 °C and then ground into a powder. In total, 15–20 g of the powder was mixed with 100 mL of methanol-water (50:50, *v*/*v*) and stirred for 72 h. It was then filtered, and methanol was removed by rotary evaporation. The stock extract was prepared as a 1000 mg/mL solution in distilled water and stored at 4 °C.

### 2.3. Fraction Recovery by HPLC Method

Reverse-phase HPLC was employed for the detection and separation of phenolic acids [30]. Elution was performed using a binary gradient system with Eluent A (0.2% phosphoric acid) and Eluent B (0.3% acetonitrile). The gradient applied for analytical separation is as follows: 0–10 min at 10% B, 10–30 min at 25% B, 30–38 min at 60% B, 38–45 min at 60% B, and 45–45.01 min at 10% B. The column temperature was maintained at 25 °C, with a flow rate of 0.6 mL/min and an injection volume of 100 µL. UV-Vis detection was performed at 203, 280, 320, and 360 nm. Calibration curves were generated from standard solutions of 5, 10, 20, 50, 100, and 200 ppm. For pre-purification, a 50% methanol solution, determined as having the highest phenolic content, was used.

### 2.4. Chitosan Nanoparticle Coating of PGPE

#### 2.4.1. Encapsulation

Chitosan nanoparticles were prepared using the ionic gelation method described by Subair et al. [31]. Chitosan was dissolved in 1% (*v*/*v*) acetic acid and stirred for 24 h at room temperature to obtain a homogeneous solution. Tween 80 was added to the mixture was stirred for 2 h at 60 °C. After cooling to room temperature, PGPE was added dropwise, maintaining a chitosan to bioactive compound ratio of 1:1.25. Finally, a 0.5% (*w*/*v*) TPP solution was added, and the mixture was stirred for an additional 30 min.

#### 2.4.2. Purification

The resulting emulsion was centrifuged at 10,000× *g* for 30 min at 4 °C, and the supernatant was discarded. The pellet was washed with a 1% (*v*/*v*) Tween 80 solution at twice the volume of the pellet to remove unbound compounds. To remove residual impurities, the nanoparticles were washed twice with deionized water, aliquoted, and stored at 4 °C.

### 2.5. Antimicrobial Susceptibility Tests

#### 2.5.1. Agar Well Diffusion Method

In the agar well diffusion method, a well with a diameter of 8 mm was used in Mueller–Hinton Agar (MHA) with a standard thickness of 4 mm [32]. Bacterial suspensions adjusted to 0.5 McFarland were spread onto the agar surface. Wells were filled with 100 μL of PGPE at concentrations of 400, 200, 100, 50, 25, and 12.5 mg/mL, while sterile distilled water served as the negative control. Each concentration was applied once per plate, and the assay was independently repeated in triplicate. After overnight incubation at 37 °C, inhibition zones were recorded. As all strains showed growth inhibition, the lowest effective concentration was subsequently determined using the minimal inhibitory concentration method.

#### 2.5.2. Minimum Inhibitory Concentration (MIC) Analysis

Pure cultures in the logarithmic growth phase were adjusted to 0.5 McFarland (1.5 × 10^8^ CFU/mL). For the MIC assay, two-fold serial dilutions of PGPE and CH-PGPE were prepared at 256, 128, 64, 32, 16, 8, 4, and 2 µg/mL, with negative and positive controls. Each concentration was tested once per run, and the assay was independently repeated twice. Following incubation, the MIC was defined as the lowest concentration showing no visible bacterial growth. CLSI breakpoints were applied, where MIC ≥ 32 µg/mL indicates resistance (R), 8–16 µg/mL intermediate susceptibility (I), and ≤4 µg/mL susceptibility (S) [33].

#### 2.5.3. Disk Diffusion Method

In the disk diffusion assay, sterile blank disks were impregnated with 400 mg/mL PGPE and CH-PGPE. Ten disks were placed on each MHA plate: eight containing the appropriate control antibiotics and two centrally positioned disks with PGPE and CH-PGPE. Bacterial suspensions adjusted to 0.5 McFarland were inoculated onto the agar surface, and disks were applied with sterile forceps. Disks were tested once per plate, and the assay was independently repeated three times. After 24 h incubation at 35 °C, inhibition zones were measured. Antimicrobial activity was interpreted according to CLSI breakpoints: ≤14 mm as resistant (R), 15–19 mm as intermediates (I), and ≥20 mm as susceptible (S) [33].

### 2.6. MTT Cytotoxicity Assay

The MTT assay was performed according to the method of Mossmann [34]. PGPE was tested at 800, 400, 200, 100, 50, and 25 μg/mL, with each concentration applied to four replicate wells, and the experiment was independently repeated three times. After 48 h of treatment, 10 μL of MTT solution (5 mg/mL) was added to each well and incubated for 4 h at 37 °C. The medium was then removed, and the resulting formazan crystals were dissolved in DMSO. The absorbance was measured at 570 nm using a microplate reader.

### 2.7. Statistical Analysis

Statistical differences were analyzed using an unpaired *t*-test. Data are presented as mean ± standard deviation (SD), and *p*-values less than 0.05 were considered statistically significant. Most experiments were conducted in triplicate.

## 3. Results

### 3.1. HPLC

The highest phenolic content was obtained using 50% methanol (4082.22 µg PUN/g), whereas 80% and 100% methanol yielded considerably lower levels (1395 µg and 1179.53 µg PUN/g, respectively). Similarly, EGCG content was maximal in 50% methanol (127.95 µg EGCG/g). Therefore, 50% methanol was selected as the extraction solvent. The identified compounds and corresponding mass spectral data are presented in Table 1.

### 3.2. Nanoparticle Characterization

**Figure 2:**
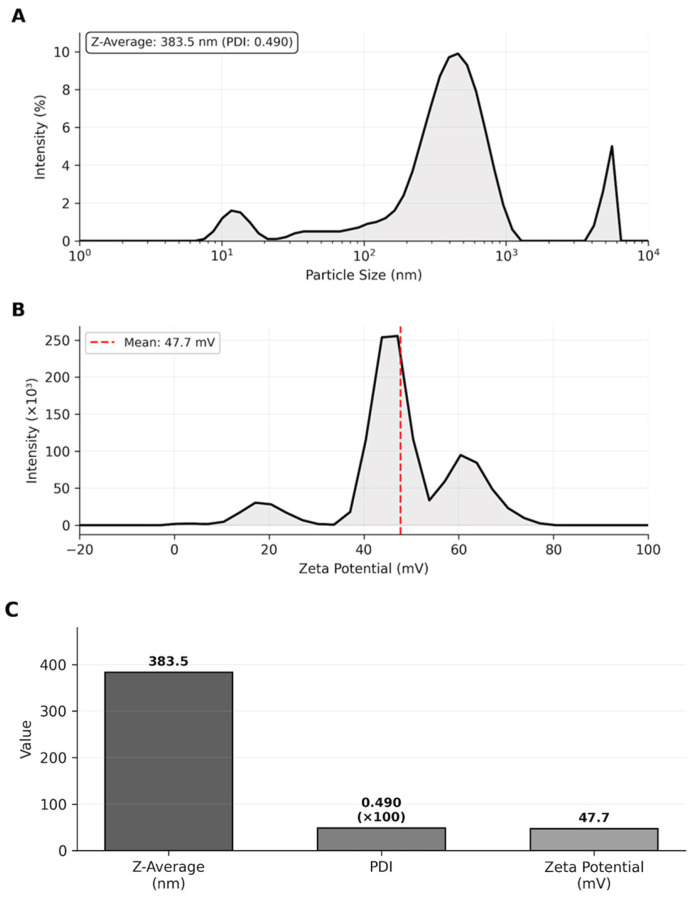
Nanoparticle characterization. (**A**) Particle size distribution by intensity showing Z-average. (**B**) Zeta potential distribution. (**C**) Summary of characterization parameters demonstrating suitable physicochemical properties for transdermal delivery.

The Z-average of 383.5 nm falls within the optimal size range (100–500 nm) for transdermal drug delivery systems, which was suitable for penetration through the stratum corneum while avoiding rapid systemic clearance. The PDI value of 0.490 indicated acceptable size distribution homogeneity. The high positive zeta potential (+47.7 mV, exceeding the ±30 mV stability threshold) ensured excellent colloidal stability through strong electrostatic repulsion between particles, preventing aggregation and maintaining long-term suspension stability (Table 2, Figure 2).

Particle size distribution by intensity showing Z-average of 383.5 nm with PDI of 0.490. Zeta potential distribution with a mean value of +47.7 mV, indicating excellent colloidal stability.

### 3.3. Antimicrobial Activity

#### 3.3.1. Agar Well Diffusion Test Results

The antimicrobial activity of PGPE was evaluated against standard ATCC reference strains following CLSI guidelines to ensure reproducibility and comparability with the literature [33]. PGPE inhibited the growth of all tested bacteria in a dose-dependent manner. Sensitivity varied among the strains: *S. aureus* showed the greatest susceptibility (32 ± 1.00 mm), whereas *P. aeruginosa* displayed the smallest inhibition zone (24 ± 1.00 mm) (Table 3).

#### 3.3.2. MIC Analysis

MIC values of PGPE and CH-PGPE were determined against clinically isolated strains. The MIC distributions for both formulations across the tested bacteria are presented in Table 4.

PGPE exhibited intermediate activity (I) against *S. aureus, E. coli*, and *K. pneumoniae*, while showing resistance (R) against *P. aeruginosa*. In contrast, CH-PGPE demonstrated consistently stronger antimicrobial activity across all strains, indicating intermediate effectiveness (I). Nanoparticle coating markedly enhanced antibacterial efficacy, reducing MIC values by approximately half for both Gram-positive and Gram-negative bacteria. Overall, CH-PGPE displayed a broader and more potent antimicrobial spectrum compared to PGPE.

#### 3.3.3. Disk Diffusion Test Results

**Figure 3:**
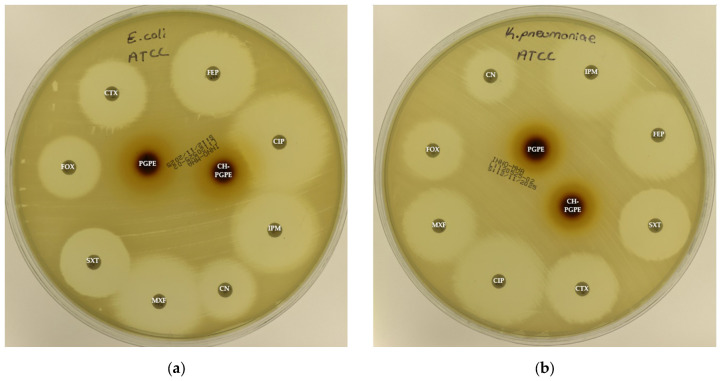

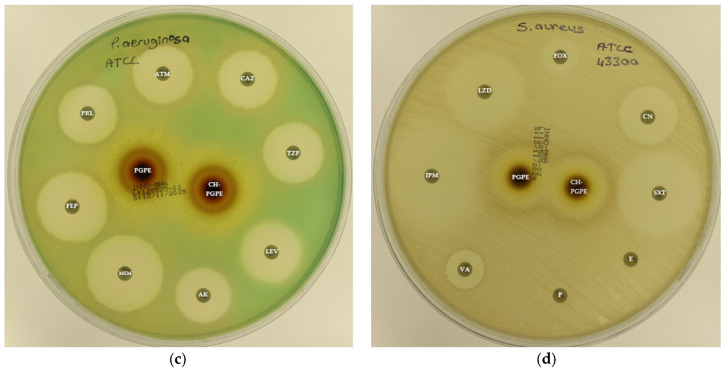
(**a**) Inhibition zones on *Escherichia coli* ATCC 25922; (**b**) Inhibition zones on *Klebsiella pneumoniae* ATCC 13883; (**c**) Inhibition zones on *Pseudomonas aeruginosa* ATCC 27853; (**d**) Inhibition zones on *Staphylococcus aureus* ATCC 43300. AK: Amikacin, ATM: Aztreonam, CAZ: Ceftazidime, CH-PGPE: Chitosan-coated *P. granatum* Peel Extract (400 mg/mL), CIP: Ciprofloxacin, CN: Gentamicin, CTX: Cefotaxime, E: Erythromycin, FEP: Cefepime, FOX: Cefoxitin, IPM: Imipenem, LEV: Levofloxacin, LZD: Linezolid, MEM: Meropenem, MXF: Moxifloxacin, P: Penicillin, PGPE: *P. granatum* Peel Extract (400 mg/mL), PRL: Piperacillin, SXT: Sulphamethoxazole/trimethoprim, TZP: Tazobactam/piperacillin, VA: Vancomycin.

**Figure 4:**
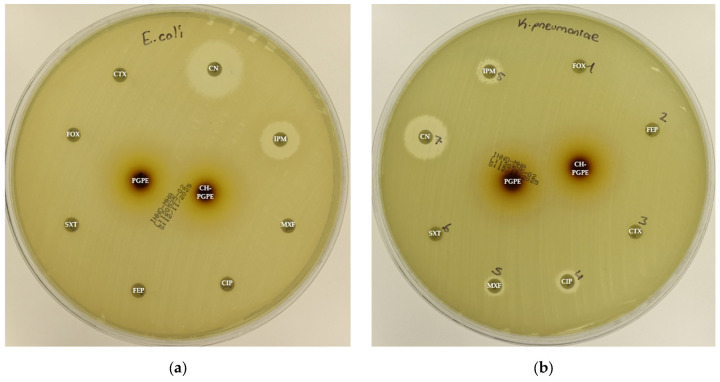

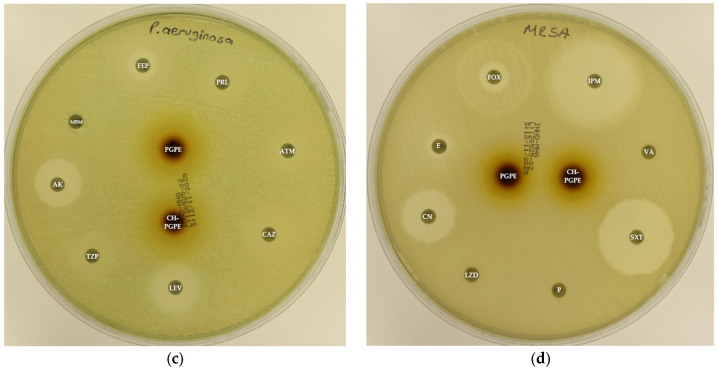
(**a**) Inhibition zones on MDR *Escherichia coli*; (**b**) Inhibition zones on MDR *Klebsiella pneumoniae*; (**c**) Inhibition zones on MDR *Pseudomonas aeruginosa;* (**d**) Inhibition zones on Methicillin-resistant *Staphylococcus aureus.* AK: Amikacin, ATM: Aztreonam, CAZ: Ceftazidime, CH-PGPE: Chitosan-coated *P. granatum* Peel Extract (400 mg/mL), CIP: Ciprofloxacin, CN: Gentamicin, CTX: Cefotaxime, E: Erythromycin, FEP: Cefepime, FOX: Cefoxitin, IPM: Imipenem, LEV: Levofloxacin, LZD: Linezolid, MEM: Meropenem, MXF: Moxifloxacin, P: Penicillin, PGPE: *P. granatum* Peel Extract (400 mg/mL), PRL: Piperacillin, SXT: Sulphamethoxazole/trimethoprim, TZP: Tazobactam/piperacillin, VA: Vancomycin.

The disk diffusion analysis was performed with both ATCC reference strains and clinical isolates of *E. coli*, *K. pneumoniae*, *P. aeruginosa*, and *S. aureus*, to demonstrate the potential clinical relevance and compare antimicrobial responses. The inhibition zones produced by the treatments are presented in Figure 3 and Figure 4. According to preliminary experiments, 400 mg/mL was identified as the optimal effective concentration of PGPE: therefore, the PGPE and CH-PGPE impregnated disks were prepared at this concentration.

**Figure 5:**
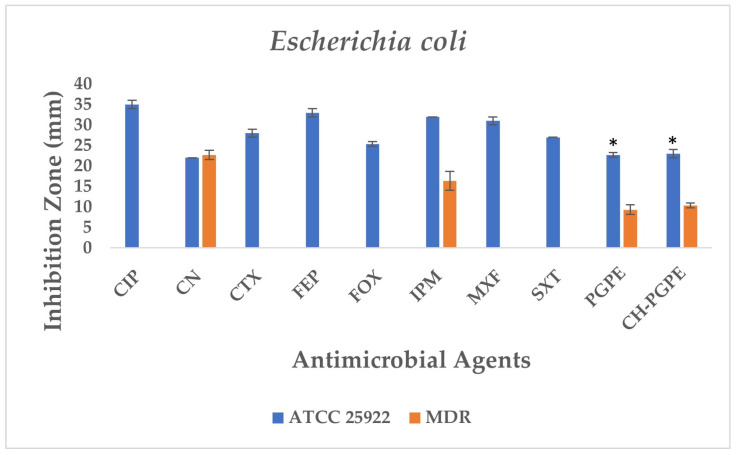
Antimicrobial activity of PGPE and CH-PGPE against *E. coli* compared to reference antibiotics. CIP: Ciprofloxacin; CN: Gentamicin; CTX: Cefotaxime; FEP: Cefepime; FOX: Cefoxitin; IPM: Imipenem; MXF: Moxifloxacin; SXT: Sulphamethoxazole/trimethoprim; PGPE: *P. granatum* Peel Extract (400 mg/mL); CH-PGPE: Chitosan-coated *P. granatum* Peel Extract (400 mg/mL); MDR: Multi-drug resistance. Results were analyzed using Student’s unpaired *t*-test in GraphPad (n = 3). * *p* < 0.05.

According to the CLSI M100 interpretive criteria for *E. coli*, the quality control strain (*E. coli* ATCC 25922) exhibited inhibition zones within the expected susceptible range for all tested antibiotics, confirming the reliability of the assay (Figure 5) [33]. In contrast, the multidrug-resistant (MDR) *E. coli* isolate showed markedly reduced or absent inhibition zones for several antibiotics. Complete resistance was observed to CIP, CTX, FEP, FOX, MXF, and SXT, with no measurable inhibition zones. The strain also exhibited resistance to IPM (32 ± 0.00 mm) and partial sensitivity to CN (22 ± 0.00 mm).

PGPE and CH-PGPE produced inhibition zones of 10 ± 1.15 mm each against the MDR strain, indicating a low but measurable antibacterial effect. In contrast, both extracts exhibited moderate activity (23 ± 0.58 mm) against the ATCC 25922 control strain, corresponding to a susceptible range. These findings confirm that while the MDR isolate was resistant to multiple conventional antibiotics, PGPE and CH-PGPE demonstrated mild inhibitory effects, suggesting that the bioactive components of *P. granatum* retain some antibacterial potential even against resistant strains. Unpaired *t*-test analysis showed that PGPE produced significantly greater inhibition in *E. coli* ATCC 22922 than in the MDR strain; the difference between the ATCC and MDR group was statistically significant (*p* < 0.05). Similarly, CH-PGPE produced stronger inhibition in the ATCC strain compared with the MDR isolate; this difference was statistically significant (*p* < 0.05).

**Figure 6:**
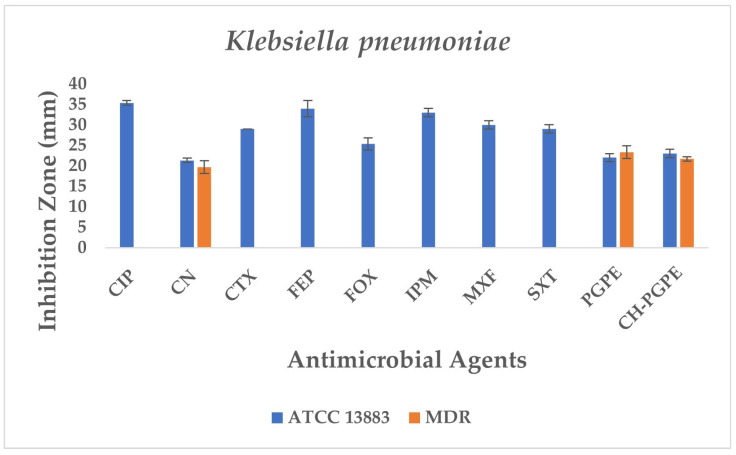
Antimicrobial activity of PGPE and CH-PGPE on *K. pneumoniae* compared to reference antibiotics. CIP: Ciprofloxacin; CN: Gentamicin; CTX: Cefotaxime; FEP: Cefepime; FOX: Cefoxitin; IPM: Imipenem; MXF: Moxifloxacin; SXT: Sulphamethoxazole/trimethoprim; PGPE: *P. granatum* Peel Extract (400 mg/mL); CH-PGPE: Chitosan-coated *P. granatum* Peel Extract (400 mg/mL); MDR: Multi-drug resistance.

The reference strain *K. pneumoniae* ATCC 13883 exhibited inhibition zones consistent with the susceptible range for all tested antibiotics (Figure 6). MDR *K. pneumoniae* isolate, however, showed a complete lack of inhibition against most antibiotics, including CIP, CTX, FEP, FOX, IPM, MXF, and SXT, indicating extensive resistance. Only CN produced a measurable zone of 18 ± 1.53 mm, which corresponds to intermediate susceptibility.

Interestingly, both PGPE and CH-PGPE demonstrated clear inhibitory activity against the MDR strain, producing zones of 23 ± 1.53 mm and 21 ± 0.58 mm, respectively. Against the susceptible ATCC 13883 strain, the inhibition zones were 23 ± 1.00 mm for PGPE and 24 ± 1.00 mm for CH-PGPE, indicating consistent antibacterial activity. These results suggest that both PGPE and CH-PGPE retained their antimicrobial potential even against highly resistant *K. pneumoniae*, with CH-PGPE exhibiting slightly reduced but comparable activity to PGPE. There is no statistically significant difference between PGPE and CH-PGPE (*p* > 0.05).

**Figure 7:**
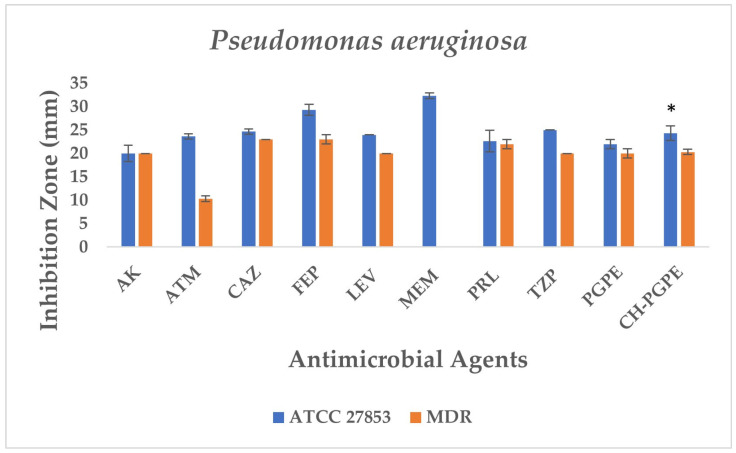
Antimicrobial activity of PGPE and CH-PGPE on *P. aeruginosa* compared to reference antibiotics. AK: Amikacin; ATM: Aztreonam; CAZ: Ceftazidime; FEP: Cefepime; LEV: Levofloxacin; MEM: Meropenem; PRL: Piperacillin; TZP: Tazobactam/piperacillin; PGPE: *P. granatum* Peel Extract (400 mg/mL); CH-PGPE: Chitosan-coated *P. granatum* Peel Extract (400 mg/mL); MDR: Multi-drug resistance. Results were analyzed using Student’s unpaired *t*-test in GraphPad (n = 3). * *p* < 0.05.

*P. aeruginosa* ATCC 27853 reference strain exhibited inhibition zones within the expected susceptible ranges for all tested antibiotics, confirming the reliability of the antimicrobial susceptibility testing procedure. In contrast, the MDR *P. aeruginosa* isolate demonstrated markedly reduced inhibition zones or complete resistance to several antibiotics (Figure 7). The strain showed no measurable inhibition against MEM, indicating carbapenem resistance, and significantly reduced susceptibility to ATM (11 ± 0.58 mm). Moderate inhibition zones were observed for AK (20 ± 0.5 mm), CAZ (23 ± 0.5 mm), FEP (24 ± 1.00 mm), LEV (20 ± 0.5 mm), PRL (21 ± 1.00 mm), and TZP (20 ± 0.58 mm), all of which fall below CLSI susceptibility breakpoints, confirming the MDR phenotype.

PGPE and CH-PGPE demonstrated measurable antibacterial activity against the MDR isolate, each producing inhibition zones of 21 mm. Against the susceptible ATCC 27853 strain, PGPE and CH-PGPE formed inhibition zones of 22 ± 1.00 mm and 24 ± 1.53 mm, respectively, suggesting consistent antibacterial potential. These findings indicate that PGPE and CH-PGPE maintain inhibitory activity even against multidrug-resistant *P. aeruginosa*, comparable to several conventional antibiotics tested in this study. According to the unpaired *t*-test results between the *P. aeruginosa* ATCC and MDR groups, the inhibitory effect of the ATCC strain was higher when CH-PGPE was applied than the MDR strain, and this difference was statistically significant (*p* < 0.05).

**Figure 8:**
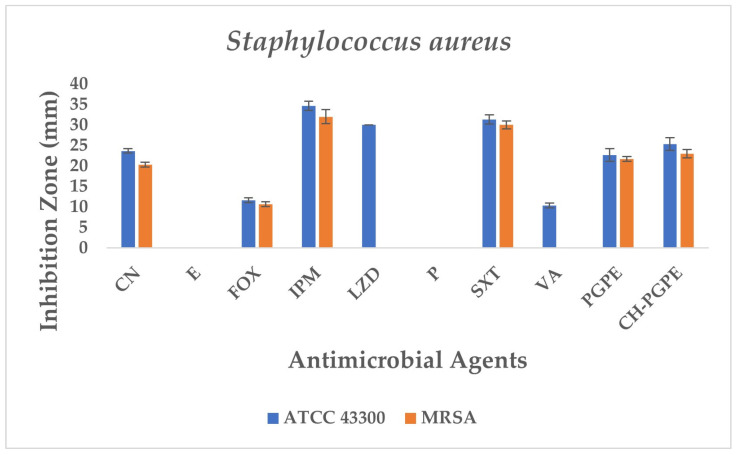
Antimicrobial activity of PGPE and CH-PGPE on *S. aureus* compared to reference antibiotics. CN: Gentamicin; E: Erythromycin; FOX: Cefoxitin; IPM: Imipenem; LZD: Linezolid; P: Penicillin; SXT: Sulphamethoxazole/trimethoprim; VA: Vancomycin; PGPE: *P. granatum* Peel Extract (400 mg/mL); CH-PGPE: Chitosan-coated *P. granatum* Peel Extract (400 mg/mL); MRSA Methicillin-resistant *S. aureus*.

*S. aureus* ATCC 43300 reference strain displayed inhibition zones within the expected susceptible ranges for most antibiotics (Figure 8). In contrast, the MRSA isolate exhibited resistance to several antibiotics, including E, P, LZD, and VA, with no measurable inhibition zones. The strain showed only weak inhibition to FOX (11 ± 0.58 mm), confirming its methicillin-resistant phenotype. CN (23 ± 0.58 mm) and SXT (31 ± 1.15 mm) displayed intermediate to susceptible activity, while IPM (34 ± 1.15 mm) produced the largest inhibition zone, indicating preserved sensitivity.

PGPE and CH-PGPE demonstrated measurable antibacterial activity against MRSA, with inhibition zones of 21 ± 0.58 mm and 23 ± 1.00 mm. Against the susceptible ATCC strain, PGPE and CH-PGPE produced inhibition zones of 22 ± 1.53 mm and 25 ± 1.53 mm, respectively. These findings indicate that both PGPE and CH-PGPE retained inhibitory effects against MRSA, with comparable or superior activity to certain conventional antibiotics tested, particularly those showing resistance in the MRSA isolate. There is no statistically significant difference between PGPE and CH-PGPE (*p* > 0.05).

### 3.4. MTT Analysis

**Figure 9:**
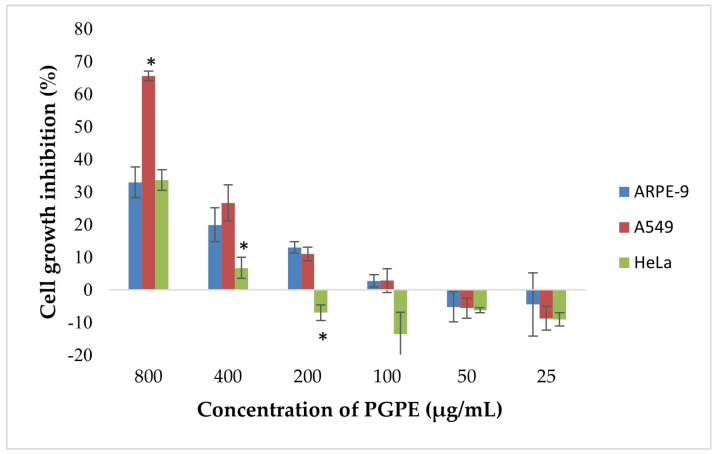
Cytotoxic effects of PGPE on human cancer and diploid cell lines. Results were analyzed using Student’s unpaired *t*-test in GraphPad (n = 4). * *p* < 0.05.

According to the results, dose-dependent inhibition of cell proliferation was observed in A549 and HeLa cells (Figure 9). The highest inhibition was detected at 800 µg/mL, reaching approximately 68% in A549 and 33.6% in HeLa cells. In contrast, ARPE-19 cells exhibited a lower inhibition rate (~30%) at the same concentration, indicating selective cytotoxicity toward cancer cells. Statistically significant differences (*p* < 0.05) in growth inhibition were observed between A549 and ARPE-19 cells at 800 and 400 µg/mL, and between HeLa and ARPE-19 at 400 µg/mL. At concentrations ≤ 200 µg/mL, growth inhibition markedly decreased and was negligible or slightly negative, particularly in ARPE-19 and HeLa cells. No significant inhibition was observed below 100 µg/mL in any of the extract cell lines.

## 4. Discussion

Previous studies provide evidence for the protective effect of *P. granatum* peel on organs in sepsis [35,36]. Sepsis is a systemic condition that develops in response to infection [37]. Therefore, the antibacterial effectiveness of *P. granatum* has been observed on microorganisms that are the most common causative agents of sepsis, which are also known to cause nosocomial infections [36]. *P. granatum* is a plant that possesses antibacterial properties and is widely used as a traditional medicine for the treatment of pathogenic bacteria [11,38]. The antimicrobial effectiveness of *P. granatum* has been tested on *Salmonella* strains, which are bacterial pathogens that cause food poisoning and gastroenteritis and have significant morbidity and mortality. It has been reported that it exhibited antibacterial activity even against *Salmonella typhimurium* (JOL 389), which has high resistance rates [38]. Additionally, it was found to be inhibitory on *Bacillus cereus*, *B. subtilis*, *B. coagulens*, *S. aureus*, *E. coli*, *P. aeruginosa*, as well as on oral pathogens *S. epidermidis*, *L. acidophilus*, *S. mutans*, and *S. salivarius* [39,40,41]. The antimicrobial activity of *P. granatum* may be related to polyphenol structures, as polyphenols can affect the bacterial cell wall, inhibit hydrolytic enzymes, interact with proteins, and disrupt the coaggregation of microorganisms [19]. Additionally, *P. granatum* is quite rich in punicalagins, which are multiple esters of gallic acid and glucose known for their antioxidant, antimicrobial, and anti-inflammatory properties [42,43,44]. However, the bioavailability of phenolic compounds in living systems is generally low. It has been shown that the use of *P. granatum*’s active ingredients together with polysaccharide-based chitosan increases its antioxidant capacity [45]. In our study, it was determined that the inhibitory effect of PGPE on Gram-positive and Gram-negative pathogenic bacteria increased with chitosan coating and exhibited significant antibacterial activity at MIC levels. Notably, after nanoparticle coating, the MIC values were reduced by half for all strains. Although there is no visibly significant difference at the macro level according to the disk diffusion results, MIC results indicate that nanoparticle coating significantly increases efficacy at the micro level. This situation shows that when phenolic components are coated with nanoparticles, their bioavailability and cellular uptake increase, thereby enhancing their antimicrobial activity.

In general, Gram-positive bacteria are considered more sensitive than Gram-negative bacteria to different antimicrobial compounds because of the differences in the structure of their cell walls [39]. Similarly, *S. aureus* was found to be more sensitive against both PGPE and CH-PGPE. MRSA is an important pathogen that is cross-resistant to most β-lactam antibiotics. When the PBP2a gene expression was analyzed by Western blot, it was confirmed by an increase in Punicalagin’s binding affinity to the cell wall peptidoglycan [19]. Chitosan coating reduced the MIC values for *S. aureus*, *E. coli*, and *K. pneumoniae* from 16 µg/mL to 8 µg/mL. *P. aeruginosa* had an MIC value of 32 µg/mL, being identified as the most resistant strain against PGPE; however, after nanoparticle coating, these values decreased to 16 µg/mL. Considering the highly drug-resistant nature of *P. aeruginosa*, it is believed that the CH-PGPE formulation could be effective even against resistant bacteria. This suggests that the nanoparticle carrier system enhances the bioavailability of phenolic compounds and their passage through the cell membrane. This increase can be explained by the more effective transport of compounds, controlled release, and enhanced interaction with microbial cells, thanks to the nanoparticle system. In the MIC analysis of pure PGPE on MDR and ATCC strains, *P. aeruginosa* MDR was detected at 20 μg/μL, *P. aeruginosa* ATCC at 30 μg/μL, and *S. aureus* ATCC and MRSA at 4 and 12 μg/μL, respectively [46]. Gosset-Erard and colleagues showed that *P. granatum* peel consists mostly of pedunculagin I and II, ellagic acid and its derivatives, and punicalagin and its isomers [47]. It has been found that the peel extract of *P. granatum* has an inhibitory effect on *S. epidermidis* (ATCC 1222) and *P. aeruginosa* (ATCC 9027), and it also provides MIC values of 0.60 µg/mL for *P. aeruginosa* (ATCC 9027), 1.2 µg/mL for *E. coli* (ATCC 25922), and 0.60 µg/mL for *S. aureus* (ATCC 6538) [47]. It has been reported that the phenolics contained in pomegranate extract, such as gallic acid, ellagic acid, and punicalagin, exert their effects by disrupting cell wall integrity and causing protein denaturation [19].

Combination with nanoparticles potentially enhances this effect in a synergistic manner. CH-PGPE, which has antibacterial potential, can reduce the increasing costs and mortality associated with treatment failures caused by resistant bacteria, serving as an alternative to expensive commercial drugs. In a study comparing the antibacterial activities of pomegranate extract and its chitosan-coated form, it was reported that nanoparticle coating led to a decrease in MRSA MIC values [48]. When inhibition zone diameters and MIC values were compared, the chitosan-coated formulation was reported to exhibit enhanced antimicrobial activity against *P. aeruginosa* [49]. PGPE and CH-PGPE were compared with various antibiotics tested against reference (ATCC) and multidrug-resistant (MDR/MRSA) bacterial strains. According to the antibiogram results, CH-PGPE demonstrated superiority over many antibiotics by showing inhibition zones ranging from 10 to 24 mm against resistant strains. While ciprofloxacin, cefepime, and meropenem were completely ineffective against MDR strains, CH-PGPE provided 10–20 mm inhibition. Additionally, it showed similar activity against *S. aureus* and MRSA when compared to tobramycin and vancomycin. These results suggest that nanoparticle-supported herbal compounds could be an effective alternative in combating antibiotic resistance. While most conventional antibiotics do not show inhibitory effects in resistant strains, CH-PGPE provided inhibition with a diameter of 10–22 mm. In Gram-positive bacteria (*S. aureus*/MRSA), CH-PGPE showed an effect close to Vancomycin and Linezolid (22 mm). While the effect of antibiotics in Gram-negative bacteria has largely been nullified in resistant strains, PGPE has retained its effect. The results indicate that PGPE and CH-PGPE exhibit high and consistent antimicrobial activity against multidrug-resistant bacteria compared to conventional antibiotics. In terms of antimicrobial activity, CH-PGPE should be considered a natural and innovative alternative against antibiotic resistance, as it competes with many conventional antibiotics and even demonstrates superior performance against resistant bacteria. Scaglione and colleagues demonstrated that pomegranate peel extract of *P. granatum* exhibits antimicrobial activity against MDR *Enterococcus faecium*, *S. aureus*, *K. pneumoniae*, *A. baumannii*, and *P. aeruginosa*, and further reported that its bactericidal effect is dose- and time-dependent, with high concentrations also providing antioxidant protection to cellular systems and red blood cell membranes [46]. When the in vitro effect on cell viability (HeLa) was evaluated, no cytotoxic effect was observed at 50–150 μg/μL range [46]. The findings of this study indicated that the compound has the strongest antiproliferative effect on A549 cells at higher concentrations (>200 µg/mL) among the three cell lines (HeLa, A549, ARPE-19) tested, with comparatively lower cytotoxicity observed in HeLa and non-cancerous ARPE-19 cells.

## 5. Conclusions

This study demonstrates that *Punica granatum* peel extract (PGPE) has notable dose-dependent antibacterial activity. Encapsulation within chitosan (CH) significantly enhances its efficacy by reducing MIC values. Both PGPE and CH-PGPE exhibited stronger or equivalent inhibition than several standard antibiotics, with pronounced effects against resistant strains. CH-PGPE showed particularly strong inhibition against resistant strains, while maintaining biocompatibility with human cells. These results indicate the potential of combining plant-derived bioactive compounds with nanotechnological systems to improve antimicrobial efficacy. The chitosan-based nanoparticle form of PGPE may serve as a promising natural alternative or adjunct in addressing antimicrobial resistance.

Further work is required to establish safety profiles, optimal dosing strategies, pharmacokinetic parameters, anti-biofilm activity, and formulation stability. Comprehensive preclinical and clinical studies, including the evaluation of purified compound metabolism and therapeutic interactions, will be essential to advance CH-PGPE toward potential clinical application as an adjuvant or combination therapy.

## Figures and Tables

**Table 1:** PGPE phenolic compounds in increasing methanol concentrations (µg/g).

Compounds	50% Methanol	80% Methanol	100% Methanol
Punicalagin isomer I	4082.22	1395.00	1179.53
Punicalagin isomer II	2048.85	835.25	539.31
Epigallocatechingallat	127.95	72.86	52.76

**Table 2:** Size, zeta potential, and polydispersity index of nanoparticles.

Formulation	Size (SD), nm	PDI (Polydispersity Index)	Zeta Potential (mV)
Nanoparticles	383.5 ± 15.2	0.490 ± 0.025	+47.7 ± 3.2
Assessment	Suitable	Acceptable	Excellent stability

**Table 3:** The inhibition zone diameters created by PGPE (mm).

PGPE	*E. coli* ATCC 25922	*K. pneumoniae* ATCC 13883	*P. aeruginosa* ATCC 27853	*S. aureus* ATCC 43300
400 mg/mL	26 ± 1.52	25 ± 0.58	24 ± 1.00	32 ± 1.00
200 mg/mL	25 ± 1.53	24 ± 0.58	23 ± 0.58	30 ± 1.53
100 mg/mL	22 ± 0.58	20 ± 0.58	20 ± 0.58	28 ± 1.53
50 mg/mL	20 ± 1.00	16 ± 1.00	18 ± 1.15	26 ± 2.08
25 mg/mL	18 ± 0.58	12 ± 1.52	16 ± 1.00	24 ± 1.15
12.5 mg/mL	16 ± 1.15	8 ± 0.58	12 ± 0.58	22 ± 1.00
Negative control	0.00	0.00	0.00	0.00

**Table 4:** MIC values of PGPE and CH-PGPE (µg/mL).

Bacterial Strains	PGPE	CH-PGPE
*Staphylococcus aureus*	16	8
*Escherichia coli*	16	8
*Klebsiella pneumoniae*	16	8
*Pseudomonas aeruginosa*	32	16

## Data Availability

The data presented in this study are available from the corresponding author upon reasonable request. No publicly archived datasets were generated or analyzed during the current study.

## Abbreviations

The following abbreviations are used in this manuscript:

AK	Amikacin
ATCC	American Type Culture Collection
ATM	Aztreonam
CAZ	Ceftazidime
CH-PGPE	Chitosan-coated *Punica granatum* peel extract
CIP	Ciprofloxacin
CLSI	Clinical and Laboratory Standard Institute
CTX	Cefotaxime
DNA	Deoxyribonucleic acid
EGCG	Epigallocatechin gallate
E	Erythromycin
FEP	Cefepime
FOX	Cefoxitin
HPLC	High Performance Liquid Chromatography
I	Intermediate
IPM	Imipenem
LEV	Levofloxacin
LZD	Linezolid
MDR	Multidrug-resistant
MEM	Meropenem
MHA	Mueller–Hinton agar
MHB	Mueller–Hinton broth
MIC	Minimum Inhibitory Concentration
MRSA	Methicillin resistant *Staphylococcus aureus*
MSSA	Methicillin-sensitive *Staphylococcus aureus*
MXF	Moxifloxacin
P	Penicillin
PGPE	*Punica granatum* peel extract
PRL	Piperacillin
R	Resistant
S	Susceptible
SXT	Sulphamethoxazole/trimethoprim
TZP	Tazobactam/piperacillin
VA	Vancomycin
WHO	World Health Organization
